# Adjunctive dexamethasone in bacterial meningitis: a meta-analysis of individual patient data

**DOI:** 10.1016/S1474-4422(10)70023-5

**Published:** 2010-03

**Authors:** Diederik van de Beek, Jeremy J Farrar, Jan de Gans, Nguyen Thi Hoang Mai, Elizabeth M Molyneux, Heikki Peltola, Tim E Peto, Irmeli Roine, Mathew Scarborough, Constance Schultsz, Guy E Thwaites, Phung Quoc Tuan, AH Zwinderman

**Affiliations:** a Department of Neurology, Centre of Infection and Immunity Amsterdam, Academic Medical Center, Amsterdam, Netherlands; b Centre for Poverty-related and Communicable Diseases, Academic Medical Center, Amsterdam, Netherlands; c Department of Clinical Epidemiology and Biostatistics, Academic Medical Center, Amsterdam, Netherlands; d Hospital for Tropical Diseases, Ho Chi Minh City, Vietnam; e Oxford University Clinical Research Unit, Ho Chi Minh City, Vietnam; f College of Medicine, University of Malawi, Blantyre, Malawi; g Helsinki University Central Hospital, Hospital for Children and Adolescents, Helsinki, Finland; h Centre for Tropical Medicine, Oxford University, Oxford, UK; i Nuffield Department of Clinical Laboratory Science, Oxford University, Oxford, UK; j Nuffield Department of Medicine, John Radcliffe Hospital, Oxford, UK; k Faculty of Health Sciences, University Diego Portales, Santiago, Chile; l Centre for Molecular Microbiology and Infection, Imperial College, London, UK

## Abstract

**Background:**

Dexamethasone improves outcome for some patients with bacterial meningitis, but not others. We aimed to identify which patients are most likely to benefit from dexamethasone treatment.

**Methods:**

We did a meta-analysis of individual patient data from the randomised, double-blind, placebo-controlled trials of dexamethasone for bacterial meningitis in patients of all ages for which raw data were available. The pre-determined outcome measures were death at the time of first follow-up, death or severe neurological sequelae at 1 month follow-up, death or any neurological sequelae at first follow-up, and death or severe bilateral hearing loss at first follow-up. Combined odds ratios (ORs) and tests for heterogeneity were calculated using conventional Mantel-Haenszel statistics. We also did exploratory analysis of hearing loss among survivors and other exploratory subgroup analyses by use of logistic regression.

**Findings:**

Data from 2029 patients from five trials were included in the analysis (833 [41·0%] aged <15 years). HIV infection was confirmed or likely in 580 (28·6%) patients and bacterial meningitis was confirmed in 1639 (80·8%). Dexamethasone was not associated with a significant reduction in death (270 of 1019 [26·5%] on dexamethasone *vs* 275 of 1010 [27·2%] on placebo; OR 0·97, 95% CI 0·79–1·19), death or severe neurological sequelae or bilateral severe deafness (42·3% *vs* 44·3%; 0·92, 0·76–1·11), death or any neurological sequelae or any hearing loss (54·2% *vs* 57·4%; 0·89, 0·74–1·07), or death or severe bilateral hearing loss (36·4% *vs* 38·9%; 0·89, 0·73–1·69). However, dexamethasone seemed to reduce hearing loss among survivors (24·1% *vs* 29·5%; 0·77, 0·60–0·99, p=0·04). Dexamethasone had no effect in any of the prespecified subgroups, including specific causative organisms, pre-dexamethasone antibiotic treatment, HIV status, or age. Pooling of the mortality data with those of all other published trials did not significantly change the results.

**Interpretation:**

Adjunctive dexamethasone in the treatment of acute bacterial meningitis does not seem to significantly reduce death or neurological disability. There were no significant treatment effects in any of the prespecified subgroups. The benefit of adjunctive dexamethasone for all or any subgroup of patients with bacterial meningitis thus remains unproven.

**Funding:**

Wellcome Trust UK.

## Introduction

The yearly incidence of bacterial meningitis is estimated to be 2·6–6·0 cases per 100 000 in Europe and might be ten times higher in less developed countries.^1^, ^2^, ^3^, ^4^ Experimental models have shown that outcome is related to the severity of the inflammatory process in the subarachnoid space, and treatment with corticosteroids results in a reduction of the inflammatory response and improved outcome.^5^, ^6^, ^7^ These findings have prompted several randomised controlled trials of corticosteroids for bacterial meningitis.^8^ Initial results suggested that the main beneficial effect of the corticosteroid dexamethasone was to reduce the risk of hearing loss in children with *Haemophilus influenzae* type b meningitis.^9^ Additional data extended the likely benefit to those with *Streptococcus pneumoniae* meningitis.^10^ In 2004, a meta-analysis of five randomised controlled trials showed that treatment with corticosteroids reduced both mortality and neurological sequelae in adults with bacterial meningitis, without detectable adverse effects.^11^ Subsequently, a Cochrane meta-analysis of data from 20 randomised controlled trials and involving 2750 people showed an overall mortality benefit and a reduction in neurological sequelae in patients treated with adjuvant corticosteroids.^8^ However, three large randomised controlled trials published after this analysis showed conflicting results.^12^, ^13^, ^14^ Adjunctive corticosteroids seem to benefit some patients with bacterial meningitis but not others, and how to select patients who are likely to benefit is unclear. Our aim was to address this question with a meta-analysis of data from five major trials for which individual patient data were available.

## Methods

### Study selection

**Figure 1:**
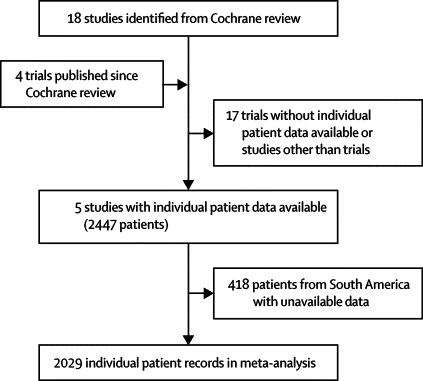
Literature search

Relevant trials were identified previously as part of a Cochrane review (figure 1).^8^ Individual patient data from five randomised, double-blind, placebo-controlled trials of dexamethasone for bacterial meningitis published since 2001 were included in the analysis;^12^, ^13^, ^14^, ^15^, ^16^ individual patient data could not be acquired from the older trials.^17^, ^18^, ^19^, ^20^, ^21^, ^22^, ^23^, ^24^, ^25^, ^26^, ^27^ The characteristics of the included studies are shown in table 1.

**Table 1:** Characteristics of the five studies included in the analysis

	**Study period**	**Patients (n)**	**Age**	**Inclusion criteria**	**Dexamethasone dose**	Empirical antibiotic*	**Primary outcome**
Europe^16^	1992–2001	301	>16 years	Clinically suspected BM plus CSF criteria	10 mg four times daily for 4 days	Intravenous amoxicillin 2 g every 4 h (77% of patients†)	Unfavourable outcome (defined by a Glasgow outcome score of 1–4) at 8 weeks
Malawi (child)^15^	1997–2001	598	2 months to 13 years	Clinically suspected BM plus CSF criteria	0·4 mg/kg twice daily for 2 days	Intravenous benzylpenicillin 200 000 IU/kg every 24 h plus chloramphenicol 100 mg/kg every 24 h	Death at 1 month
Vietnam^13^	1996–2005	429	>14 years	Clinically suspected BM plus CSF criteria	0·4 mg/kg twice daily for 4 days	Intravenous ceftriaxone 2 g every 12 h	Death at 1 month
Malawi (adult)^14^‡	2002–2005	465	>15 years	Clinically suspected BM plus CSF criteria	16 mg twice daily for 4 days	Intravenous or intramuscular ceftriaxone 2 g every 12 h	Death at 1 month
South America^12^§	1996–2003	236	2 months to 16 years	Clinically suspected BM plus CSF or blood criteria	0·15 mg/kg four times daily for 2 days	Intravenous ceftriaxone 80–100 mg/kg every 24 h	Death, severe neurological sequelae, or audiological sequelae at hospital discharge

BM=bacterial meningitis.

* Dexamethasone was given before or with the first dose of per-protocol parenteral antibiotic in all five studies.

† 23% of patients received other antibiotic treatment.

‡ 2×2 design with patients randomly assigned to dexamethasone or placebo and to intravenous or intramuscular ceftriaxone.

§ 2×2 design with patients randomly assigned to dexamethasone plus glycerol, dexamethasone plus placebo, placebo plus glycerol, or placebo plus placebo; patients assigned to receive glycerol with either dexamethasone or placebo were excluded from the individual patient data meta-analysis; data from this trial were analysed as two strata according to randomisation schedule.

The study from South America used a 2×2 design to randomly assign children with bacterial meningitis to dexamethasone plus glycerol, dexamethasone plus placebo, glycerol plus placebo, or placebo plus placebo.^12^ Data were available from children who were assigned dexamethasone plus placebo or placebo only but not from those who were given glycerol. During the study, the randomisation schedule was altered from a ratio of two dexamethasone per three placebo (randomisation schedule 1) to one dexamethasone per one placebo (randomisation schedule 2). Therefore, analyses from this study were stratified according to randomisation schedule. The study in Malawian adults used a 2×2 design to randomly assign patients to dexamethasone or placebo and to intravenous or intramuscular ceftriaxone.^14^ In all studies, patients were enrolled on the basis of clinically suspected bacterial meningitis and CSF criteria. All the studies used computer-generated randomisation to allocate patients to dexamethasone or placebo. Treatment concealment was adequate in all studies.

### Definitions and outcome measures

The members of the study group met in October, 2006, and September, 2007, to discuss data sharing and the analysis plan, including the definitions of subgroups, which were specified before the data were collated, the final database created, and the analysis started. The principal investigators provided the raw data, which were checked by a statistician (PQT). Inconsistencies and outlying data were clarified with the principal investigators and resolved from their raw data before the analysis.

15 data fields for each patient were selected for the analyses. The dataset included prognostic factors for unfavourable outcome and potential modifiers of the treatment effect of dexamethasone, such as antibiotic treatment before admission, HIV infection, and malnutrition.^1^, ^3^ Definitions were agreed during the two study-group meetings. Values for continuous variables were reassigned into categories. Exposure to antibiotics before randomisation was defined by administration of effective oral or intravenous antibiotics within 48 h before the first dose of study drug was received. Malnutrition was defined by individual investigators: patients who were not assessed were categorised according to the local prevalence of malnutrition. HIV tests were not done on every patient and an assessment was made of the likelihood of HIV infection based on local epidemiology. All untested Malawian adults were defined as likely to be HIV positive. No assumption was made for untested Malawian children. All other untested adults or children were defined as likely to be HIV negative. Impairment of consciousness was categorised by use of the Glasgow coma scale or the Blantyre coma score (table 2). The causative pathogen was defined by CSF microscopy, CSF or blood culture, PCR, or latex agglutination.

**Table 2:** Baseline characteristics of patients included in the analysis

	**Europe**^16^**(n=301)**	**Malawi (child)**^15^**(n=598)**	**Vietnam**^13^**(n=429)**	**Malawi (adult)**^14^**(n=465)**	**South America**^12^		**Total (n=2029)**	**Dexamethasone (n=1019)**	**Placebo (n=1010)**
					Randomisation schedule 1 (n=126)	Randomisation schedule 2 (n=110)			
**Age (years)**									
<5	0	429	0	0	117	90	636	316	320
5–15	1	168	0	2	9	17	197	99	98
16–55	198	0	322	447	0	0	967	490	477
>55	102	0	106	16	0	0	224	112	112
Unknown	0	1	1	0	0	3	5	2	3
**Sex**									
Men	169 (56%)	337 (56%)	315 (73%)	230 (50%)	73 (58%)	63 (57%)	1187 (58%)	601 (59%)	586 (58%)
**Symptoms <48 h**									
Yes	233 (77%)	266 (44%)	121 (28%)	121 (26%)	91 (72%)	93 (84%)	925 (46%)	471 (46%)	454 (45%)
Unknown	2 (1%)	2 (0·3%)	2 (0·5%)	5 (1%)	15 (12%)	9 (8%)	35 (2%)	17 (2%)	18 (2%)
**Prior antibiotic exposure**									
Yes	5 (2%)	127 (21%)	238 (55%)	123 (26%)	46 (36%)	35 (32%)	574 (28%)	281 (28%)	293 (29%)
Unknown	0	0	0	0	14 (11%)	11 (10%)	25 (1%)	9 (1%)	16 (2%)
**Malnutrition**									
Yes	..	307 (52%)	46 (11%)	..	6 (5%)	7 (6%)	366 (18%)	187 (18%)	179 (18%)
No	..	288 (48%)	375 (87%)	..	59 (47%)	41 (37%)	763 (38%)	380 (37%)	383 (38%)
Not assessed (likely yes)	..	..	8 (2%)	..	61 (48%)	62 (56%)	476 (23%)	237 (23%)	239 (24%)
Not assessed (likely no)	301	3 (0·5%)	..	..	..	..	424 (21%)	215 (21%)	209 (21%)
HIV infection*									
Positive/tested	0/0	157/459 (34%)	3/429 (1%)	389/434 (90%)	0/0	0/0	549/1322 (42%)	269/663 (41%)	280/659 (42%)
**Heart rate**									
≥120 beats per min	42 (14%)	513 (86%)	58 (14%)	118 (25%)	75 (60%)	56 (51%)	862 (42%)	430 (42%)	432 (43%)
Unknown	2 (1%)	8 (1%)	12 (3%)	8 (2%)	5 (4%)	7 (6%)	42 (2%)	19 (2%)	23 (2%)
**Level of consciousness**†									
Normal	103 (34%)	219 (37%)	147 (34%)	84 (18%)	23 (18%)	17 (16%)	593 (29%)	288 (28%)	305 (30%)
Mild impairment	102 (34%)	168 (28%)	145 (34%)	162 (35%)	57 (45%)	65 (59%)	699 (34%)	349 (34%)	350 (35%)
Moderate impairment	68 (23%)	125 (21%)	88 (20%)	129 (28%)	25 (20%)	17 (16%)	452 (22%)	239 (23%)	213 (21%)
Severe impairment	28 (9%)	84 (14%)	46 (11%)	90 (19%)	16 (13%)	11 (10%)	275 (14%)	137 (13%)	138 (14%)
Unknown	0	2 (0·3%)	3 (1%)	0	5 (4%)	0	10 (0·5%)	6 (0·6%)	4 (0·4%)
**Haemoglobin**									
<10 g/dL	7 (2%)	373 (62%)	14 (3%)	181 (39%)	90 (71%)	73 (66%)	738 (36%)	367 (36%)	371 (37%)
Unknown	0	70 (12%)	36 (8%)	31 (7%)	6 (5%)	3 (3%)	148 (7%)	67 (7%)	79 (8%)
**CSF white cell count (cells per μL)**									
0–99	8 (3%)	22 (4%)	7 (2%)	93 (20%)	7 (6%)	3 (3%)	140 (7%)	76 (7%)	64 (6%)
100–999	44 (15%)	172 (29%)	95 (22%)	186 (40%)	40 (32%)	27 (24%)	564 (28%)	286 (28%)	280 (28%)
1000–9999	166 (55%)	258 (43%)	253 (59%)	158 (34%)	55 (44%)	47 (43%)	937 (46%)	450 (44%)	487 (48%)
>10 000	78 (26%)	144 (24%)	72 (17%)	20 (4%)	16 (13%)	19 (17%)	349 (17%)	187 (18%)	162 (16%)
Unknown	5 (2%)	2 (0·3%)	2 (0·5%)	8 (2%)	8 (6%)	14 (13%)	39 (2%)	22 (2%)	17 (2%)
**CSF glucose (mg/dL)**‡									
≤20	169 (56%)	475 (79%)	179 (42%)	332 (71%)	64 (51%)	61 (56%)	1280 (63%)	648 (64%)	632 (62%)
>20	122 (40%)	31 (5%)	248 (58%)	72 (16%)	53 (42%)	46 (42%)	572 (28%)	284 (28%)	288 (29%)
Unknown	10 (3%)	92 (15%)	2 (0·5%)	61 (13%)	9 (7%)	3 (3%)	177 (9%)	87 (8%)	90 (9%)
**CSF protein (mg/dL)**‡									
≥250	195 (65%)	412 (69%)	191 (45%)	341 (73%)	23 (18%)	33 (30%)	1195 (59%)	588 (58%)	607 (60%)
<250	97 (32%)	26 (4%)	227 (53%)	33 (7%)	77 (61%)	73 (66%)	533 (26%)	283 (28%)	250 (25%)
Unknown	9 (3%)	160 (27%)	11 (2%)	91 (20%)	26 (20%)	4 (4%)	301 (15%)	148 (14%)	153 (15%)
**Bacteria on CSF microscopy**									
Yes	215 (71%)	427 (71%)	249 (57%)	263 (57%)	74 (59%)	69 (63%)	1297 (64%)	641 (63%)	656 (65%)
Unknown	0	0	0	15 (3%)	34 (27%)	24 (22%)	73 (4%)	38 (4%)	35 (4%)
**Causative pathogen**									
*Streptococcus pneumoniae*	112 (37%)	238 (40%)	77 (18%)	275 (59%)	22 (18%)	35 (32%)	759 (37%)	383 (38%)	376 (37%)
*Neisseria meningitidis*	115 (38%)	67 (11%)	29 (7%)	20 (4%)	4 (3%)	4 (4%)	239 (12%)	122 (12%)	117 (12%)
*Haemophilus influenzae*	4 (1%)	170 (28%)	8 (2%)	3 (0·6%)	66 (52%)	46 (42%)	297 (15%)	135 (13%)	162 (16%)
*Streptococcus suis*	..	..	137 (32%)	..	..	..	137 (7%)	72 (7%)	65 (6%)
Other aerobic gram negative bacilli	3 (1%)	38 (6%)	21 (5%)	22 (5%)	3 (2%)	1 (1%)	88 (4%)	51 (5%)	37 (4%)
Other	27 (9%)	11 (2%)	68 (16%)	5 (1%)	3 (2%)	5 (4%)	119 (6%)	52 (5%)	67 (7%)
Definitely not bacterial meningitis	0	0	11 (3%)	38 (8%)	1 (1%)	0	50 (2·5%)	28 (3%)	22 (2%)
Unknown (probable bacterial meningitis)	40 (13%)	74 (12%)	78 (18%)	102 (22%)	27 (21%)	19 (17%)	340 (17%)	176 (17%)	164 (16%)
Bacterial diagnosis confirmed microbiologically	261 (87%)	524 (88%)	340 (78%)	325 (70%)	98 (78%)	91 (83%)	1639 (81%)	815 (80%)	824 (81%)
**Treatment allocation**									
Allocated to dexamethasone treatment	157 (52%)	305 (51%)	215 (50%)	233 (50%)	50 (40%)	59 (54%)	1019 (50%)	..	..

Data are number (%).

* HIV serostatus was not available in patients in the European or South American trials; patients in these trials were assumed to be HIV negative. In the Vietnam trial, four untested patients were assumed to be HIV negative and in the Malawi adult trial, 31 untested patients were assumed to be HIV positive. In the Malawi paediatric trial, 139 (23%) patients were not tested and no assumption was made about their serostatus. Positive values only include patients tested and not those assumed to be positive.

† Glasgow coma scale is categorised in adults as 15=normal, 11–14=mild impairment, 8–10=moderate impairment, 3–7=severe impairment. Blantyre coma score is categorised as 5=normal, 3–4=mild impairment, 2=moderate impairment, 0–1=severe impairment.

‡ The use of urine reagent strips in the trials from Malawi provided semi-quantitative estimates of CSF glucose and protein. Protein and glucose concentrations were measured with a urine dipstick (Multistix 8SG Bayer), which provided colour-coded results of (protein/glucose) negative (0/0 mg/dL); trace (<30/100 mg/dL); 1+ (31–100/101–250 mg/dL); 2+ (101–300/251–500 mg/dL); 3+ (301–2000/501–1000 mg/dL); 4+ (>2000/>1000 mg/dL).

The predetermined outcome measures were death at the time of first follow-up; death or severe neurological sequelae (including severe bilateral hearing loss) at 1 month follow-up; death or any neurological sequelae (including any degree of hearing loss) at first follow-up; and death or severe bilateral hearing loss at first follow-up. The number of studies that contributed to each outcome is shown by degrees of freedom (df=number of studies minus 1). Additionally, as part of a post-hoc exploratory analysis and to analyse every possible endpoint of interest, we analysed hearing loss of any degree among survivors. The severity of neurological sequelae in the adult studies was defined using the Glasgow outcome score or the modified Rankin scale.^28^, ^29^ In the paediatric studies, severe neurological disability was defined as blindness, quadraparesis, hydrocephalus requiring a shunt, or severe psychomotor retardation. Hearing loss was categorised as moderate or severe according to definitions used in the individual studies.

### Statistical analysis

All analyses were stratified according to study site (including two strata from the South American study) to account for any possible centre effect, including differences in mortality between centres. If appropriate, analyses were also stratified according to the baseline variable of interest. Combined odds ratios (ORs) and tests for heterogeneity were calculated using conventional Mantel-Haenszel statistics. We also used exploratory analyses with logistic regression. The main purpose of the analysis was to establish whether dexamethasone had a differential effect in different subgroups of patients; hence, heterogeneity between the subgroups (*I*^2^ values) with significance levels were calculated for each subgroup analysis. Tests for heterogeneity were calculated without allowing for multiple comparisons, to increase the sensitivity of detecting any evidence of between-subgroup heterogeneity. To maximise the power of finding significant heterogeneity, missing values were removed, except where indicated, from the subgroup analyses. A continuity correction was made for zero events. Significance tests, with the appropriate degrees of freedom, were calculated to test for possible heterogeneity between studies for each subgroup analysis.

To calculate the combined ORs for death from studies included in the Cochrane reviews but not otherwise included in the present study, results available from the published literature were combined by use of conventional Mantel-Haenszel statistics. Calculation of combined ORs and 95% CIs, tests of heterogeneity between studies, and logistic regression analyses were done by use of STATA version 10.

### Role of the funding source

The study sponsors had no role in the study design, collection, analysis, and interpretation of the data, or the decision to submit the manuscript for publication. T E Peto had full access to all data in the study. All authors approved and were responsible for submission of the manuscript.

## Results

The baseline characteristics were similar in placebo and dexamethasone groups within the five studies (table 2). 1019 (50·2%) patients received dexamethasone and 1010 (49·8%) patients received placebo. 833 (41·1%) patients were less than 15 years old, of whom 415 received dexamethasone and 418 received placebo. 1196 adults (aged ≥15 years) were included, of whom 604 (50·5%) received dexamethasone and 592 (49·5%) received placebo. The ages of five patients were unknown.

HIV co-infection was confirmed in 549 (41·5%) of 1322 patients tested, of whom 391 (71·4%) were adults and 158 (28·8%) were children. An HIV test was not done in 707 (34·8%) patients but, on the basis of epidemiological risk, was judged likely to be positive in 31 untested adults from Malawi and negative in adults from Europe and children from South America. No assumption was made about 139 untested children from Malawi. In total, 286 confirmed or likely HIV-infected patients received dexamethasone and 294 received placebo.

The diagnosis of bacterial meningitis was microbiologically confirmed in 1639 (80·8%) patients and was most frequently caused by *S pneumoniae* (759 cases), *H influenzae* (297 cases), and *Neisseria meningitidis* (239 cases). The most common causative bacteria per study were as follows: Europe, *N meningitis* (38%);^16^ Malawi (children), *S pneumoniae* (40%);^15^ Vietnam, *Streptococcus suis* (32%);^13^ Malawi (adults), *S pneumoniae* (59%);^14^ and South America, *H influenzae* (47%).^12^ Mortality in the placebo groups differed substantially between studies: 15% in Europe, 31% in Malawian children, 12% in Vietnam, 53% in Malawian adults, and 16% in South America.

Dexamethasone was not associated with a significant reduction in death, death or severe neurological sequelae (including severe bilateral hearing loss), death or any neurological sequelae (including any hearing loss), or death or severe bilateral hearing loss, if all patients were included in the analysis (table 3). However, hearing loss (of any severity) in survivors was less common in the dexamethasone group (162 [24·1%] of 672 *vs* 195 [29·5%] of 660; OR 0·77 [95% CI 0·60–0·99], p=0·04).

**Table 3:** Primary endpoints for each study and for all patients assigned to steroid therapy

	**Europe**^16^	**Malawi (child)**^15^	**Vietnam**^13^	**Malawi (adult)**^14^	**South America**^12^		**Overall**	**Events/total (%)**		**Test for heterogeneity**		
					Randomisation schedule 1	Randomisation schedule 2		Dexamethasone	Placebo	χ^2^ (5 df)	p	*I*^2^
Death	0·44 (0·20–0·96, 0·03)	0·96 (0·70–1·40, 0·96)	0·82 (0·45–1·51, 0·53)	1·16 (0·80–1·67, 0·43)	1·46 (0·63–3·37, 0·37)	0·74 (0·27–2·00, 0·55)	0·97 (0·79–1·19, 0·75)	270/1019 (26·5%)	275/1010 (27·2%)	6·5	0·26	22·7%
Death or severe neurological sequelae or bilateral severe deafness	0·60 (0·34–1·11, 0·07)	1·20 (0·87–1·66, 0·28)	0·75 (0·48–1·17, 0·20)	1·02 (0·69–1·50, 0·93)	0·74 (0·35–1·55, 0·42)	0·74 (0·33–1·67, 0·52)	0·92 (0·76–1·11, 0·39)	424/1003 (42·3%)	439/992 (44·3%)	6·5	0·26	23·2%
Death or any neurological sequelae or any hearing loss	0·49 (0·28–0·84, 0·01)	1·02 (0·74–1·42, 0·89)	0·81 (0·55–1·18, 0·27)	1·03 (0·67–1·56, 0·91)	1·29 (0·60–2·77, 0·51)	0·84 (0·39–1·79, 0·65)	0·89 (0·74–1·07, 0·23)	541/999 (54·2%)	567/988 (57·4%)	7·1	0·22	29·3%
Death or severe bilateral hearing loss	0·55 (0·31–0·99, 0·04)	1·03 (0·73–1·45, 0·86)	0·64 (0·38–1·08, 0·09)	1·08 (0·73–1·58, 0·70)	1·07 (0·49–2·32, 0·87)	0·70 (0·29–1·69, 0·43)	0·89 (0·73–1·69, 0·23)	343/942 (36·4%)	363/934 (38·9%)	6·2	0·28	19·8%
Any hearing loss in survivors	0·75 (0·34–1·67, 0·48)	0·80 (0·51–1·28, 0·35)	0·77 (0·49–1·21, 0·26)	0·80 (0·44–1·45, 0·45)	0·59 (0·21–1·65, 0·31)	0·81 (0·30–2·14, 0·66)	0·77 (0·60–0·99, 0·04)	162/672 (24·1%)	195/660 (29·5%)	0·3	1·00	0·0%

Data are OR (95% CI, p value) unless otherwise stated. OR values below 1 suggest a beneficial effect of steroids.

**Figure 2:**
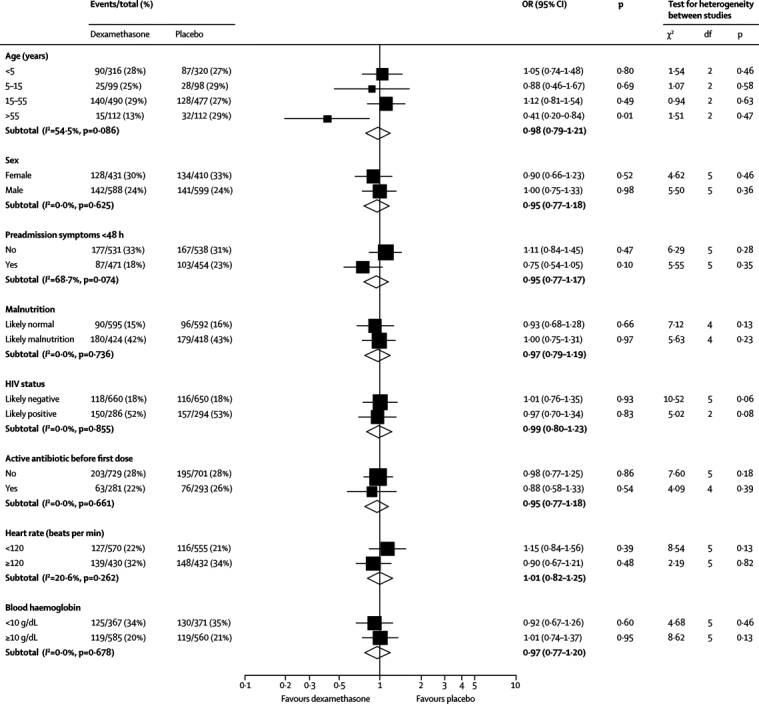
Subgroup analyses for death BM=bacterial meningitis. OR=odds ratio.

**Figure 3:**
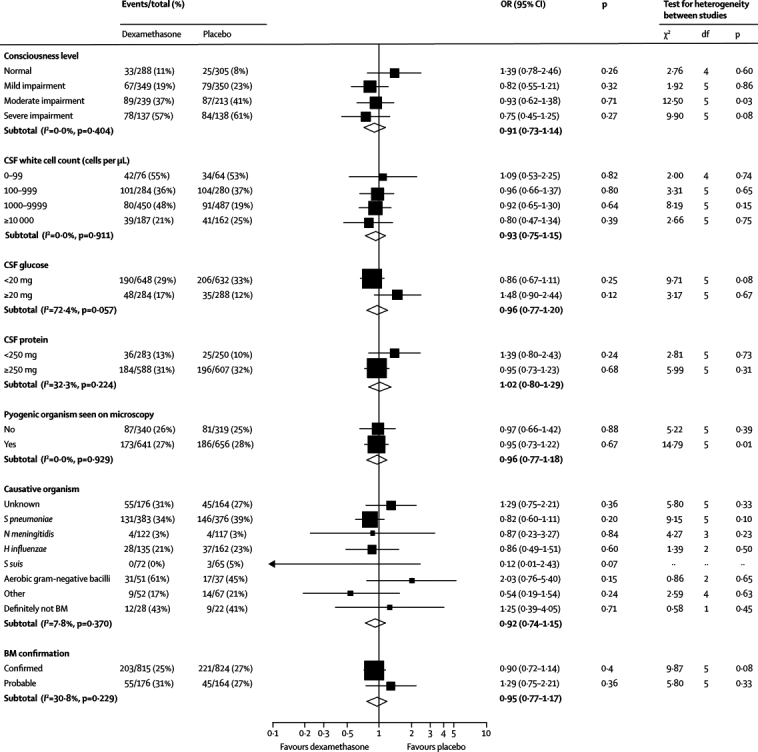
Subgroup analyses for death BM=bacterial meningitis. OR=odds ratio.

The subgroup analyses for all outcome measures are shown in Figure 2, Figure 3, and the webappendix. Duration of symptoms before treatment, severity of coma at start of treatment, whether dexamethasone was given before or after antibiotics, and HIV infection status did not significantly influence treatment response. Dexamethasone was more effective in patients aged older than 55 years in analyses of death (OR 0·41 [95% CI 0·20–0·84], p=0·01), death or severe neurological sequelae (OR 0·53 [0·30–0·84], p=0·03), and death or any neurological sequelae (OR 0·56 [0·31–1·00], p=0·05). However, there was no clear evidence of heterogeneity between the different age groups (death, χ^2^=6·9, 3 df, p=0·07, *I*^2^ 54·5%; death or severe neurological sequelae, χ^2^=6·6, 3 df, p=0·09, *I*^2^=53·4%; death or any neurological sequelae, χ^2^=4·4, 3 df, p=0·23, *I*^2^=30·3%). Further exploratory analyses, using age as a continuous variable, did not show any consistent interaction between age and a treatment effect (data not shown). There was also no effect in a post-hoc analysis that restricted the study to patients treated with ceftriaxone (webappendix).

The data were explored to identify evidence of heterogeneity between the studies. 23 subgroups were explored, each with five different endpoints. In patients with moderate CNS impairment on admission, there was some evidence of heterogeneity between three of the five endpoints. In the subgroup of patients with moderate CNS impairment on admission, there was evidence of benefit in death or severe neurological sequelae or bilateral hearing loss in the European study (OR 0·19 [95% CI 0·04–0·82], p=0·01), but also evidence of harm in the study of children in Malawi (OR 3·70 [1·36–10·08], p=0·006). However, no evidence of heterogeneity was observed in patients with either no or little CNS impairment or with severe CNS impairment. Overall, there was no evidence of any difference in outcome for any of the CNS subgroups in any of the five endpoints. The effect of HIV was explored by adjustment with logistic regression analysis and also by studying only patients with proven HIV status. However, HIV status did not have an effect on dexamethasone treatment outcome (webappendix). We further explored the relation between age, HIV status, and dexamethasone treatment effect (table 4). In HIV-negative adults, dexamethasone was associated with a reduction in death or severe neurological sequelae, including severe bilateral hearing loss (OR 0·68 [95% CI 0·48–0·95], p=0·02), death or any neurological sequelae, including any hearing loss (OR 0·67 [0·50–0·91], p=0·01), and death or severe bilateral hearing loss (OR 0·61 [0·42–0·89], p=0·01). However, this effect of dexamethasone was not present in HIV-negative children, or in HIV-positive children and adults.

**Table 4:** Exploratory analyses of the influence of age and HIV infection on the treatment effect of dexamethasone

	**HIV negative**		**HIV positive**		**Overall**	**Test for heterogeneity**		
	Adult	Child	Adult	Child		χ^2^ (3 df)	p	*I*^2^
Death	0·66 (0·42–1·02, 0·06)	1·43 (0·96–2·12, 0·07)	1·19 (0·81–1·75, 0·36)	0·54 (0·28–1·03, 0·06)	0·99 (0·80–1·23, 0·99)	10·7	0·01	72·0%
Death or severe neurological sequelae or bilateral severe deafness	0·68 (0·48–0·95, 0·02)	1·09 (0·77–1·55, 0·62)	1·10 (0·73–1·66, 0·67)	0·77 (0·36–1·66, 0·44)	0·90 (0·73–1·10, 0·29)	4·8	0·19	37·4%
Death or any neurological sequelae or any hearing loss	0·67 (0·50–0·91, 0·01)	1·09 (0·77–1·56, 0·62)	1·15 (0·73–1·82, 0·54)	0·77 (0·35–1·71, 0·53)	0·88 (0·72–1·07, 0·18)	6·0	0·11	50·1%
Death or severe bilateral hearing loss	0·61 (0·42–0·89, 0·01)	1·16 (0·80–1·67, 0·43)	1·13 (0·75–1·70, 0·55)	0·62 (0·30–1·29, 0·20)	0·89 (0·72–1·09, 0·26)	8·1	0·04	28·5%
Any hearing loss in survivors	0·76 (0·52–1·13, 0·17)	0·67 (0·42–1·07, 0·09)	0·87 (0·46–1·63, 0·66)	1·09 (0·37–3·19, 0·87)	0·77 (0·59–0·99, 0·06)	0·9	0·83	0·0%

Data are OR (95% CI, p value) unless otherwise stated. Adults were defined as ≥15 years. HIV negative includes patients who tested negative or were likely to be negative. HIV positive includes those who tested positive or were likely to be positive.

Gastrointestinal bleeding was reported in all studies: 13 (1·3%) of 1021 patients on dexamethasone and 19 (1·9%) of 1014 patients on placebo (p=0·14). Hyperglycaemia and infection by herpes simplex virus and varicella zoster virus were reported in some but not all studies.^4^, ^13^, ^16^ Hyperglycaemia was recorded by the trials in Malawian and European adults and was significantly associated with dexamethasone treatment (79 of 390 [20·3%] on dexamethasone *vs* 60 of 376 [16·0%] on placebo; p=0·02). Neither infection with herpes simplex virus (labial infection in all) nor infection with varicella zoster virus were significantly associated with dexamethasone treatment.

**Figure 4:**
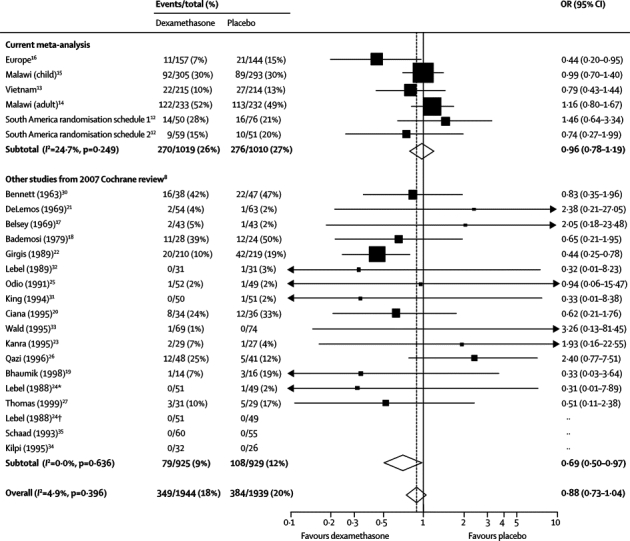
Effect of adjunctive dexamethasone therapy on death Trials included in the rest of this study^12^, ^13^, ^14^, ^15^, ^16^ and other studies^17^, ^18^, ^19^, ^20^, ^21^, ^22^, ^23^, ^24^, ^25^, ^26^, ^27^, ^30^, ^31^, ^32^, ^33^, ^34^, ^35^ included in the Cochrane systematic review^8^ are shown. OR=odds ratio. *Study 1 in Lebel.^24^ †Study 2 in Lebel.^24^

Dexamethasone did not significantly affect mortality in a combined analysis with the data from other studies included in the Cochrane analysis^8^ (OR 0·88 [95% CI 0·73–1·04], p=0·14; figure 4).^17^, ^18^, ^19^, ^20^, ^21^, ^22^, ^23^, ^24^, ^25^, ^26^, ^27^, ^30^, ^31^, ^32^, ^33^, ^34^, ^35^ 349 (18·0%) of 1944 patients who received dexamethasone died, compared with 384 (19·8%) of 1939 patients who received placebo. There was no evidence of significant heterogeneity between the trials.

## Discussion

The aim of this analysis was to establish whether any subgroups of patients with acute bacterial meningitis might benefit from adjunctive dexamethasone and thereby explain any differences between individual trial results. Extensive exploration of 15 prespecified subgroups did not show robust evidence that a particular subgroup would benefit. The apparent benefit in adults aged over 55 years might have occurred by chance. However, it is unclear whether it is more likely to have occurred by chance than the findings of no benefit in other subgroups.

This analysis of 2029 patients from five trials showed that treatment with adjunctive dexamethasone did not significantly reduce mortality, neurological disability, or severe hearing loss in bacterial meningitis. Combination of these results with those from older published trials, for which the raw data were not obtainable, did not show any evidence that dexamethasone was significantly effective in reducing these outcomes overall. However, a post-hoc analysis on the incidence of deafness among survivors suggested that adjunctive dexamethasone treatment reduced the rate of hearing loss (OR 0·77 [95% CI 0·60–0·99; p=0·04), irrespective of whether patients had received antibiotics before dexamethasone treatment. The use of adjunctive dexamethasone treatment was not associated with an increased risk of adverse events.

Factors previously considered relevant to the decision to start dexamethasone treatment in patients with suspected or proven bacterial meningitis could not explain differences in results between the five trials. These factors include duration of symptoms before treatment, severity of impaired consciousness at start of treatment, whether dexamethasone was given before or after antibiotics, and HIV infection status.^7^, ^36^, ^37^, ^38^, ^39^ Because the results of the prespecified analysis failed to show any significant heterogeneity, extensive post-hoc analyses were done with the inclusion of an additional deafness endpoint. Such analyses are usually considered unreliable, particularly if no statistical allowance is made for multiple comparisons, because of the high chance of a false-positive result. However, the extra analyses were undertaken to allow the identification of subgroups of interest for further possible study. These exploratory post-hoc analyses suggested a possible overall effect on deafness among survivors and on death and severe neurological sequelae in the subgroup of HIV-negative adults (OR 0·68 [95% CI 0·54–0·99], p=0·02). This apparent treatment effect ceased to be significant after adjustment for multiple comparisons.

This meta-analysis is, as are all meta-analyses, limited by the possibility that more heterogeneity exists between the studies than has been identified. If such heterogeneity were to exist, combining the studies would be inappropriate. Formal tests for heterogeneity between studies and between subgroups failed to show any convincing evidence of heterogeneity. However, such tests are insensitive and could miss important effects. We have therefore explored the data exhaustively for relevant subgroups of patients that could reveal possible causes of heterogeneity, although little such evidence was found.

On the basis of previous meta-analyses,^9^, ^10^ the administration of dexamethasone to children with *H influenzae* type b meningitis before the start of antibiotic therapy is thought to reduce the incidence of deafness. However, we found no evidence of a benefit of adjunctive dexamethasone in all children or in any subgroup of children with this infection.

In summary, these data indicate that patients with bacterial meningitis neither benefit from nor are harmed by treatment with adjunctive dexamethasone. Despite an individual patient data meta-analysis of more than 2000 patients, we have been unable to determine conclusively whether a subgroup of patients might benefit. To establish with certainty whether dexamethasone has a place in the treatment of adult patients with bacterial meningitis, a large multinational randomised controlled trial would be necessary. This represents a formidable challenge and one that is not likely to be met for many years. In the meantime, we suggest the benefit of adjunctive dexamethasone for all or any subgroup of patients with bacterial meningitis remains unproven and there is little support for its routine use in the treatment of this disease.

## Web Extra Material

Supplementary webappendix

## References

[1] van de Beek D, de Gans J, Spanjaard L, Weisfelt M, Reitsma JB, Vermeulen M (2004). Clinical features and prognostic factors in adults with bacterial meningitis. N Engl J Med.

[2] van de Beek D, de Gans J, Tunkel AR, Wijdicks EF (2006). Community-acquired bacterial meningitis in adults. N Engl J Med.

[3] Saez-Llorens X, McCracken GH (2003). Bacterial meningitis in children. Lancet.

[4] Scarborough M, Thwaites GE (2008). The diagnosis and management of acute bacterial meningitis in resource-poor settings. Lancet Neurol.

[5] Scheld WM, Dacey RG, Winn HR, Welsh JE, Jane JA, Sande MA (1980). Cerebrospinal fluid outflow resistance in rabbits with experimental meningitis: alterations with penicillin and methylprednisolone. J Clin Invest.

[6] Tauber MG, Khayam-Bashi H, Sande MA (1985). Effects of ampicillin and corticosteroids on brain water content, cerebrospinal fluid pressure, and cerebrospinal fluid lactate levels in experimental pneumococcal meningitis. J Infect Dis.

[7] van de Beek D, Weisfelt M, de Gans J, Tunkel AR, Wijdicks EF (2006). Drug insight: adjunctive therapies in adults with bacterial meningitis. Nat Clin Pract Neurol.

[8] van de Beek D, de Gans J, McIntyre P, Prasad K (2007). Corticosteroids for acute bacterial meningitis. Cochrane Database Syst Rev.

[9] Havens PL, Wendelberger KJ, Hoffman GM, Lee MB, Chusid MJ (1989). Corticosteroids as adjunctive therapy in bacterial meningitis: a meta-analysis of clinical trials. Am J Dis Child.

[10] McIntyre PB, Berkey CS, King SM (1997). Dexamethasone as adjunctive therapy in bacterial meningitis: a meta-analysis of randomized clinical trials since 1988. JAMA.

[11] van de Beek D, de Gans J, McIntyre P, Prasad K (2004). Steroids in adults with acute bacterial meningitis: a systematic review. Lancet Infect Dis.

[12] Peltola H, Roine I, Fernandez J (2007). Adjuvant glycerol and/or dexamethasone to improve the outcomes of childhood bacterial meningitis: a prospective, randomized, double-blind, placebo-controlled trial. Clin Infect Dis.

[13] Nguyen TH, Tran TH, Thwaites G (2007). Dexamethasone in Vietnamese adolescents and adults with bacterial meningitis. N Engl J Med.

[14] Scarborough M, Gordon SB, Whitty CJ (2007). Corticosteroids for bacterial meningitis in adults in sub-Saharan Africa. N Engl J Med.

[15] Molyneux EM, Walsh AL, Forsyth H (2002). Dexamethasone treatment in childhood bacterial meningitis in Malawi: a randomised controlled trial. Lancet.

[16] de Gans J, van de Beek D (2002). Dexamethasone in adults with bacterial meningitis. N Engl J Med.

[17] Belsey MA, Hoffpauir CW, Smith MH (1969). Dexamethasone in the treatment of acute bacterial meningitis: the effect of study design on the interpretation of results. Pediatrics.

[18] Bademosi O, Osuntokun BO (1979). Prednisolone in the treatment of pneumococcal meningitis. Trop Geograph Med.

[19] Bhaumik S, Behari M (1998). Role of dexamethasone as adjunctive therapy in acute bacterial meningitis in adults. Neurol India.

[20] Ciana G, Parmar N, Antonio C, Pivetta S, Tamburlini G, Cuttini M (1995). Effectiveness of adjunctive treatment with steroids in reducing short-term mortality in a high-risk population of children with bacterial meningitis. J Trop Pediatr.

[21] deLemos RA, Haggerty RJ (1969). Corticosteroids as an adjunct to treatment in bacterial meningitis: a controlled clinical trial. Pediatrics.

[22] Girgis NI, Farid Z, Mikhail IA, Farrag I, Sultan Y, Kilpatrick ME (1989). Dexamethasone treatment for bacterial meningitis in children and adults. Pediatr Infect Dis J.

[23] Kanra GY, Ozen H, Secmeer G, Ceyhan M, Ecevit Z, Belgin E (1995). Beneficial effects of dexamethasone in children with pneumococcal meningitis. Pediatr Infect Dis J.

[24] Lebel MH, Freij BJ, Syrogiannopoulos GA (1988). Dexamethasone therapy for bacterial meningitis. Results of two double-blind, placebo-controlled trials. N Engl J Med.

[25] Odio CM, Faingezicht I, Paris M (1991). The beneficial effects of early dexamethasone administration in infants and children with bacterial meningitis. N Engl J Med.

[26] Qazi SA, Khan MA, Mughal N (1996). Dexamethasone and bacterial meningitis in Pakistan. Arch Dis Child.

[27] Thomas R, Le TY, Bouget J (1999). Trial of dexamethasone treatment for severe bacterial meningitis in adults: adult Meningitis Steroid Group. Intensive Care Med.

[28] Jennett B, Bond M (1975). Assessment of outcome after severe brain damage. Lancet.

[29] UK-TIA Study Group (1988). United Kingdom transient ischaemic attack (UK-TIA) aspirin trial: interim results. BMJ.

[30] Bennett IL, Finland M, Hamburger M, Kass EH, Lepper M, Waisbren N (1963). The effectiveness of hydrocortisone in the management of severe infections. JAMA.

[31] King SM, Law B, Langley JM, Heurtler H, Bremner D, Wang EE (1994). Dexamethasone therapy for bacterial meningitis: better never than late?. Can J Infect Dis.

[32] Lebel MH, Hoyt J, Waagner DC, Rollins NK, Finitzo T, McCracken GH (1989). Magnetic resonance imaging and dexamethasone therapy for bacterial meningitis. Am J Dis Child.

[33] Wald ER, Kaplan SL, Mason EO (1995). Dexamethasone therapy for children with bacterial meningitis. Pediatrics.

[34] Kilpi T, Peltola H, Jauhiainen T (1995). Oral glycerol and intravenous dexamethasone in preventing neurologic and audiological sequelae of childhood bacterial-meningitis. Pediatr Infect Dis J.

[35] Schaad UB, Lips U, Gnehm HE, Blumberg A, Heinzer I, Wedgwood J (1993). Dexamethasone therapy for bacterial meningitis in children. Lancet.

[36] van de Beek D, de Gans J (2006). Dexamethasone in adults with community-acquired bacterial meningitis. Drugs.

[37] Tunkel AR, Hartman BJ, Kaplan SL (2004). Practice guidelines for the management of bacterial meningitis. Clin Infect Dis.

[38] Fitch MT, van de Beek D (2008). Drug insight: steroids in CNS infectious diseases—new indications for an old therapy. Nat Clin Pract Neurol.

[39] Greenwood BM (2007). Corticosteroids for acute bacterial meningitis. N Engl J Med.

