# Supplementary material for: Adjunctive dexamethasone in bacterial meningitis: a meta-analysis of individual patient data
Source: Lancet Neurol. 2010 Mar;9(3):254–63. doi: 10.1016/S1474-4422(10)70023-5 (PMC2835871; doi:10.1016/S1474-4422(10)70023-5)
Supplement: Supplementary webappendix [file mmc1.pdf]

THE LANCET Neurology

Supplementary webappendix

This webappendix formed part of the original submission and has been peer reviewed. We post it as supplied by the authors.

Supplement to: van de Beek D, Farrar JJ, de Gans J, et al. Adjunctive dexamethasone in bacterial meningitis: a meta-analysis of individual patient data. *Lancet Neurol* 2010; published online February 4. DOI:1016/ S1474-4422(10)70023-5.

Webfigure 1a: Subgroup analysis for death or severe neurological sequelae (including severe bilateral hearing loss)

	Events/total (%)		OR (95% CI)	p	Test for heterogeneity between studies		
	Dexamethasone	Placebo			χ^2	df	p
**Age (years)**							
<5	166/315 (53%)	172/320 (54%)	0·93 (0·68–1·29)	0·67	0·86	2	0·65
5–15	49/99 (49%)	41/96 (43%)	1·54 (0·85–2·77)	0·15	0·47	2	0·79
15–55	175/477 (37%)	176/462 (38%)	0·92 (0·68–1·24)	0·59	1·06	2	0·59
>55	34/112 (30%)	50/111 (45%)	0·53 (0·30–0·95)	0·03	0·21	2	0·90
**Subtotal (I^2=53·4%, p=0·092)**			**0·92 (0·76–1·11)**				
**Sex**							
Female	191/421 (45%)	207/400 (52%)	0·78 (0·59–1·05)	0·10	5·99	5	0·31
Male	233/582 (40%)	232/591 (39%)	1·01 (0·79–1·30)	0·93	5·12	5	0·40
**Subtotal (I^2=43·4%, p=0·184)**			**0·90 (0·75–1·09)**				
**Preadmission symptoms <48 h**							
No	261/519 (50%)	264/529 (50%)	1·03 (0·80–1·34)	0·80	6·38	5	0·27
Yes	155/468 (33%)	167/446 (37%)	0·79 (0·59–1·06)	0·12	6·14	5	0·29
**Subtotal (I^2=43·7%, p=0·183)**			**0·92 (0·76–1·11)**				
**Malnutrition**							
Likely normal	177/592 (30%)	200/592 (34%)	0·84 (0·65–1·08)	0·17	4·83	4	0·31
Likely malnutrition	247/411 (60%)	239/400 (60%)	1·04 (0·78–1·38)	0·78	4·80	4	0·31
**Subtotal (I^2=16·8%, p=0·273)**			**0·92 (0·76–1·12)**				
**HIV status**							
Likely negative	213/657 (32%)	233/649 (36%)	0·85 (0·67–1·08)	0·19	8·05	5	0·15
Likely positive	184/273 (67%)	188/277 (68%)	1·00 (0·70–1·44)	1·00	1·13	2	0·57
**Subtotal (I^2=0·0%, p=0·461)**			**0·89 (0·73–1·09)**				
**Active antibiotic before first dose**							
No	309/718 (43%)	304/688 (44%)	0·91 (0·73–1·14)	0·41	4·80	5	0·44
Yes	110/276 (40%)	126/289 (44%)	0·94 (0·66–1·34)	0·74	5·51	4	0·24
**Subtotal (I^2=0·0%, p=0·879)**			**0·92 (0·76–1·11)**				
**Heart rate (beats per min)**							
<120	175/558 (31%)	189/540 (35%)	0·85 (0·65–1·11)	0·23	6·24	5	0·28
≥120	241/426 (57%)	237/429 (55%)	1·05 (0·79–1·38)	0·75	2·69	5	0·75
**Subtotal (I^2=12·9%, p=0·284)**			**0·94 (0·78–1·14)**				
**Blood haemoglobin**							
<10 g/dL	194/354 (55%)	206/364 (57%)	0·91 (0·67–1·23)	0·54	4·99	5	0·42
≥10 g/dL	188/582 (32%)	189/549 (34%)	0·93 (0·71–1·20)	0·57	9·04	5	0·11
**Subtotal (I^2=0·0%, p=0·915)**			**0·92 (0·76–1·12)**				

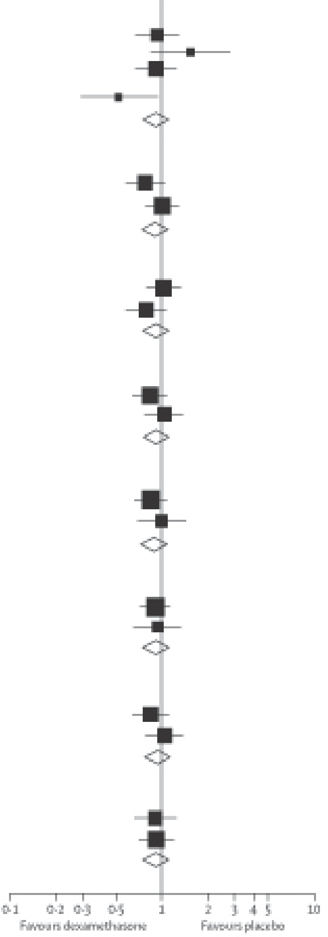

OR=odds ratio.

Webfigure 1b: Subgroup analysis for death or severe neurological sequelae (including severe bilateral hearing loss)

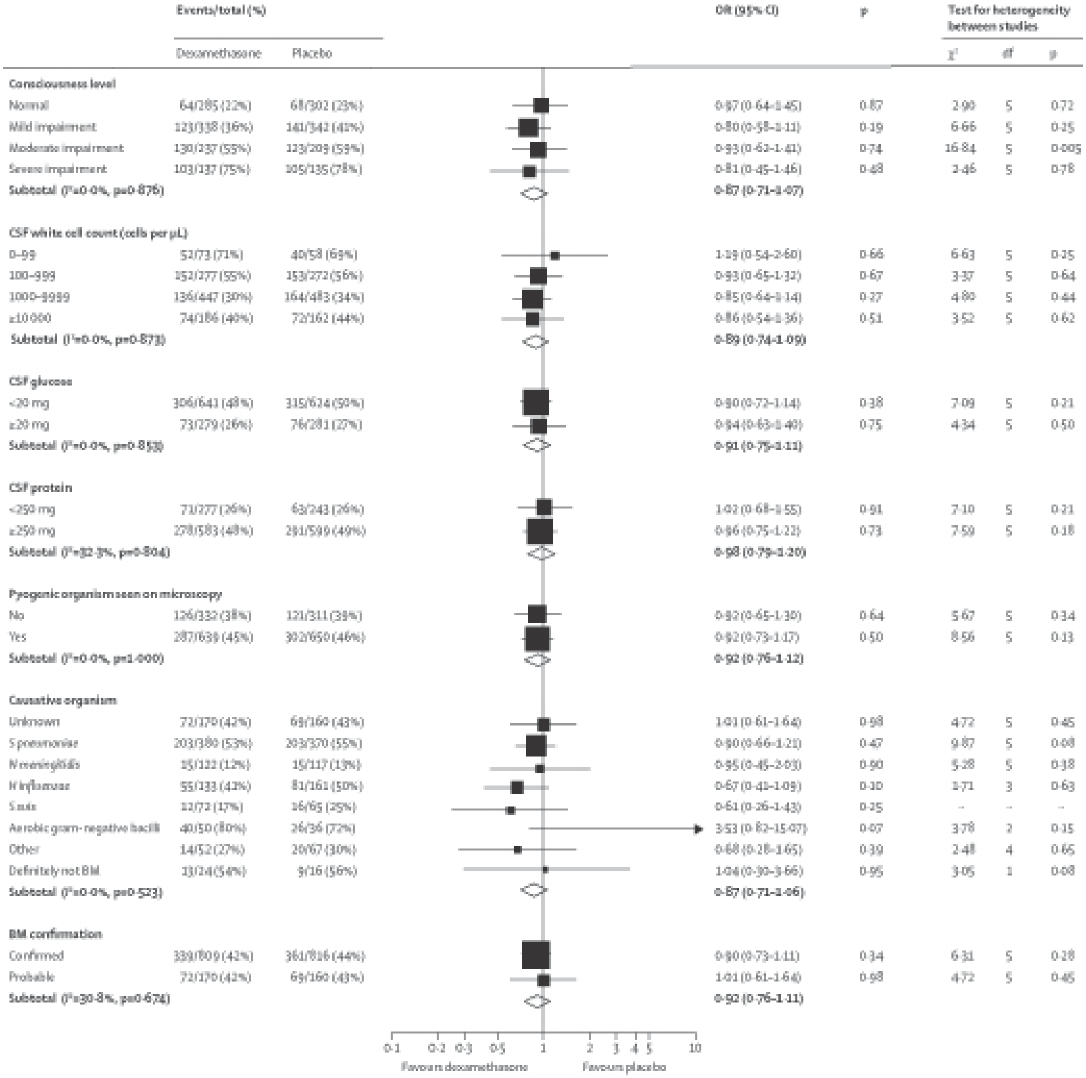

BM=bacterial meningitis. OR=odds ratio.

Webfigure 2a: Subgroup analysis for death or any neurological sequelae (including any hearing loss)

	Events/total (%)		OR (95% CI)	p	Test for heterogeneity between studies		
	Dexamethasone	Placebo			χ^2	df	p
**Age (years)**							
<5	195/313 (62%)	202/320 (63%)	0·97 (0·70–1·35)	0·87	1·65	2	0·44
5–15	54/99 (55%)	50/96 (52%)	1·28 (0·71–2·29)	0·41	0·58	2	0·75
15–55	236/473 (50%)	249/458 (54%)	0·84 (0·63–1·11)	0·22	2·63	2	0·27
>55	55/112 (49%)	65/111 (59%)	0·56 (0·31–1·00)	0·05	0·97	2	0·62
**Subtotal (I^2=30·3%, p=0·231)**			**0·88 (0·73–1·07)**				
**Sex**							
Female	240/420 (57%)	252/400 (63%)	0·79 (0·58–1·06)	0·11	5·45	5	0·36
Male	301/579 (52%)	315/587 (54%)	0·94 (0·74–1·19)	0·58	3·25	5	0·66
**Subtotal (I^2=0·0%, p=0·375)**			**0·88 (0·73–1·06)**				
**Preadmission symptoms <48 h**							
No	323/517 (62%)	339/528 (64%)	0·93 (0·71–1·20)	0·56	4·93	4	0·29
Yes	206/466 (44%)	217/443 (49%)	0·82 (0·62–1·08)	0·16	3·21	5	0·67
**Subtotal (I^2=0·0%, p=0·518)**			**0·88 (0·72–1·06)**				
**Malnutrition**							
Likely normal	250/589 (42%)	288/588 (49%)	0·78 (0·62–0·99)	0·04	5·85	4	0·21
Likely malnutrition	291/410 (71%)	279/400 (70%)	1·07 (0·79–1·44)	0·68	0·48	4	0·98
**Subtotal (I^2=62·3%, p=0·104)**			**0·88 (0·73–1·06)**				
**HIV status**							
Likely negative	305/654 (47%)	334/645 (52%)	0·82 (0·66–1·03)	0·09	7·91	5	0·16
Likely positive	207/272 (76%)	210/277 (76%)	1·03 (0·70–1·52)	0·89	1·25	2	0·53
**Subtotal (I^2=0·0%, p=0·317)**			**0·87 (0·73–1·05)**				
**Active antibiotic before first dose**							
No	383/714 (54%)	377/684 (55%)	0·93 (0·75–1·16)	0·54	8·45	5	0·13
Yes	152/276 (55%)	181/289 (63%)	0·75 (0·53–1·06)	0·11	4·58	4	0·33
**Subtotal (I^2=5·7%, p=0·303)**			**0·87 (0·73–1·05)**				
**Heart rate (beats per min)**							
<120	248/554 (45%)	275/537 (51%)	0·79 (0·61–1·01)	0·06	4·90	5	0·43
≥120	283/426 (66%)	276/429 (64%)	1·10 (0·82–1·47)	0·53	6·04	5	0·30
**Subtotal (I^2=64·7%, p=0·093)**			**0·91 (0·75–1·10)**				
**Blood haemoglobin**							
<10 g/dL	232/353 (66%)	240/364 (66%)	0·99 (0·72–1·35)	0·93	2·95	5	0·71
≥10 g/dL	262/579 (45%)	269/545 (49%)	0·86 (0·67–1·10)	0·23	8·04	5	0·15
**Subtotal (I^2=0·0%, p=0·491)**			**0·91 (0·75–1·10)**				

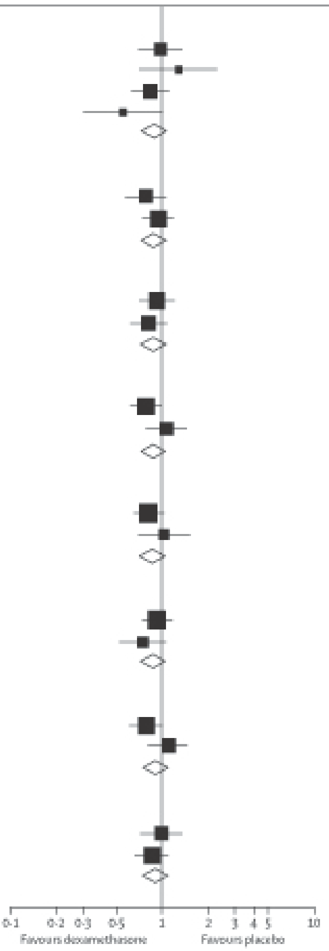

OR=odds ratio.

Webfigure 2b: Subgroup analysis for death or any neurological sequelae (including any hearing loss)

	Events/total (%) Dexamethasone	Events/total (%) Placebo	OR (95% CI)	p	Test for heterogeneity between studies χ^2	df	p
**Consciousness level**							
Normal	97/283 (34%)	100/299 (33%)	1·02 (0·72–1·46)	0·90	2·11	5	0·83
Mild impairment	173/337 (51%)	202/342 (59%)	0·74 (0·54–1·01)	0·06	2·83	5	0·73
Moderate impairment	158/236 (67%)	144/209 (69%)	0·96 (0·62–1·48)	0·85	12·61	5	0·03
Severe impairment	108/137 (79%)	119/134 (89%)	0·44 (0·22–0·87)	0·02	6·30	5	0·28
**Subtotal (I^2=45·7%, p=0·137)**			**0·83 (0·68–1·01)**				
**CSF white cell count (cells per μL)**							
0–99	59/73 (81%)	45/58 (78%)	1·32 (0·54–3·25)	0·54	3·33	5	0·65
100–999	183/277 (66%)	180/272 (66%)	1·01 (0·70–1·45)	0·97	0·96	5	0·97
1000–9999	191/445 (43%)	238/479 (50%)	0·76 (0·58–0·99)	0·04	3·32	5	0·65
≥10 000	96/184 (52%)	90/162 (56%)	0·92 (0·59–1·44)	0·72	4·58	5	0·47
**Subtotal (I^2=0·0%, p=0·472)**			**0·87 (0·72–1·05)**				
**CSF glucose**							
<20 mg	378/639 (59%)	389/622 (63%)	0·88 (0·70–1·11)	0·28	8·89	5	0·11
≥20 mg	114/277 (41%)	123/279 (44%)	0·89 (0·63–1·27)	0·53	2·70	5	0·75
**Subtotal (I^2=0·0%, p=0·958)**			**0·88 (0·73–1·07)**				
**CSF protein**							
<250 mg	115/275 (42%)	111/241 (46%)	0·88 (0·62–1·27)	0·50	9·49	5	0·09
≥250 mg	338/581 (58%)	362/597 (61%)	0·89 (0·70–1·14)	0·37	7·27	5	0·20
**Subtotal (I^2=0·0%, p=0·959)**			**0·89 (0·72–1·08)**				
**Pyogenic organism seen on microscopy**							
No	159/331 (48%)	148/309 (48%)	0·95 (0·69–1·33)	0·78	10·61	5	0·06
Yes	364/636 (57%)	398/648 (62%)	0·85 (0·67–1·07)	0·16	7·06	5	0·22
**Subtotal (I^2=0·0%, p=0·589)**			**0·88 (0·73–1·07)**				
**Causative organism**							
Unknown	83/170 (49%)	83/159 (52%)	0·89 (0·56–1·42)	0·61	8·94	5	0·11
S pneumoniae	239/379 (63%)	241/369 (65%)	0·88 (0·65–1·20)	0·43	8·95	5	0·11
N meningitidis	17/120 (14%)	24/115 (21%)	0·65 (0·33–1·29)	0·21	3·88	5	0·57
H influenzae	77/133 (58%)	99/161 (61%)	0·87 (0·54–1·40)	0·56	4·19	3	0·52
S suis	44/72 (61%)	45/65 (69%)	0·70 (0·34–1·43)	0·32	–	–	–
Aerobic gram-negative bacilli	41/50 (82%)	30/36 (83%)	1·27 (0·30–5·32)	0·75	5·11	1	0·02
Other	23/52 (44%)	35/67 (52%)	0·67 (0·31–1·44)	0·30	4·48	4	0·34
Definitely not BM	17/23 (74%)	10/16 (63%)	1·64 (0·39–6·93)	0·50	1·78	1	0·18
**Subtotal (I^2=0·0%, p=0·932)**			**0·84 (0·70–1·03)**				
**BM confirmation**							
Confirmed	441/806 (55%)	474/813 (58%)	0·88 (0·72–1·08)	0·22	4·56	5	0·47
Probable	83/170 (49%)	83/159 (52%)	0·89 (0·56–1·42)	0·61	8·94	5	0·11
**Subtotal (I^2=0·0%, p=0·965)**			**0·88 (0·73–1·06)**				

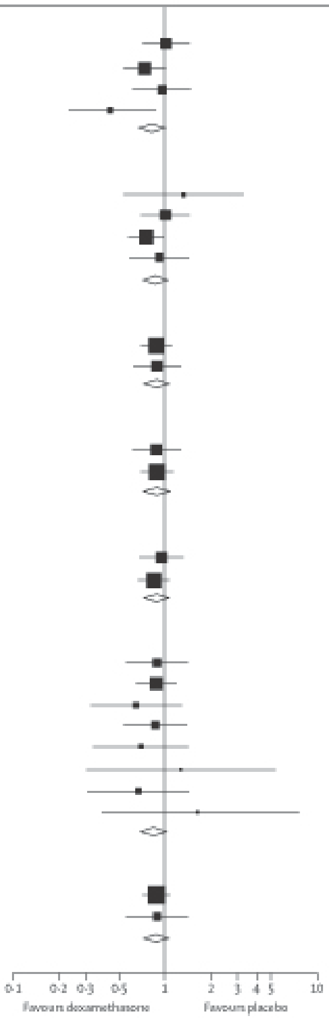

BM=bacterial meningitis. OR=odds ratio.

Webfigure 3a: Subgroup analysis for death or severe bilateral hearing loss

	Events/total (%)		OR (95% CI)	p	Test for heterogeneity between studies		
	Dexamethasone	Placebo			χ^2	df	p
**Age (years)**							
<5	119/282 (42%)	129/294 (44%)	0·89 (0·64–1·25)	0·50	0·76	2	0·68
5–15	42/87 (48%)	36/83 (43%)	1·42 (0·75–2·67)	0·28	0·55	2	0·76
15–55	158/463 (34%)	154/443 (35%)	0·95 (0·69–1·29)	0·73	2·01	2	0·37
>55	24/108 (22%)	44/111 (40%)	0·45 (0·25–0·84)	0·01	0·03	2	0·98
**Subtotal (I^2=57·8%, p=0·068)**			**0·89 (0·73–1·09)**				
**Sex**							
Female	158/398 (40%)	174/380 (46%)	0·76 (0·56–1·03)	0·07	5·38	5	0·37
Male	185/544 (34%)	189/553 (34%)	0·97 (0·74–1·26)	0·80	5·50	5	0·36
**Subtotal (I^2=28·4%, p=0·237)**			**0·87 (0·71–1·07)**				
**Preadmission symptoms <48 h**							
No	208/480 (43%)	214/494 (43%)	0·98 (0·75–1·29)	0·90	4·87	5	0·43
Yes	129/445 (29%)	143/424 (34%)	0·77 (0·57–1·04)	0·09	4·24	5	0·52
**Subtotal (I^2=26·6%, p=0·243)**			**0·88 (0·72–1·08)**				
**Malnutrition**							
Likely normal	134/558 (24%)	158/565 (27%)	0·81 (0·62–1·07)	0·13	4·39	4	0·36
Likely malnutrition	209/384 (54%)	205/369 (56%)	0·97 (0·73–1·30)	0·85	5·92	4	0·21
**Subtotal (I^2=0·0%, p=0·374)**			**0·88 (0·72–1·08)**				
**HIV status**							
Likely negative	164/629 (26%)	180/619 (29%)	0·84 (0·65–1·09)	0·20	8·11	5	0·15
Likely positive	168/267 (63%)	173/270 (64%)	0·97 (0·68–1·38)	0·86	2·90	2	0·29
**Subtotal (I^2=0·0%, p=0·520)**			**0·88 (0·72–1·09)**				
**Active antibiotic before first dose**							
No	260/676 (38%)	257/650 (40%)	0·93 (0·73–1·17)	0·53	4·17	5	0·08
Yes	79/258 (31%)	100/268 (37%)	0·76 (0·51–1·12)	0·16	5·35	4	0·77
**Subtotal (I^2=0·0%, p=0·388)**			**0·88 (0·72–1·09)**				
**Heart rate (beats per min)**							
<120	152/537 (28%)	159/520 (31%)	0·89 (0·67–1·19)	0·43	9·92	5	0·08
≥120	186/388 (48%)	192/395 (49%)	0·94 (0·70–1·25)	0·66	2·52	5	0·77
**Subtotal (I^2=0·0%, p=0·793)**			**0·91 (0·75–1·12)**				
**Blood haemoglobin**							
<10 g/dL	153/329 (47%)	170/338 (50%)	0·82 (0·60–1·13)	0·23	3·26	5	0·66
≥10 g/dL	156/552 (28%)	160/527 (30%)	0·91 (0·69–1·20)	0·48	7·75	5	0·17
**Subtotal (I^2=0·0%, p=0·627)**			**0·87 (0·71–1·07)**				

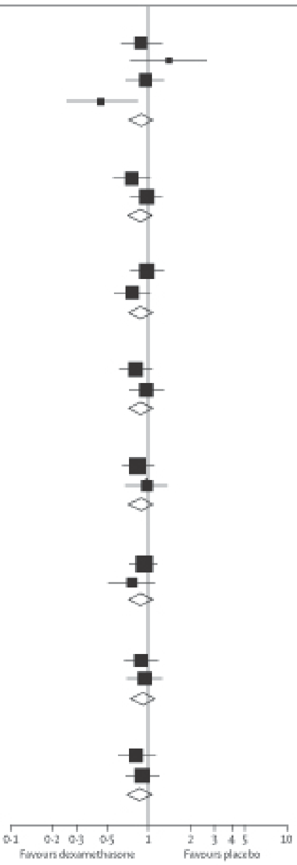

OR=odds ratio.

Webfigure 3b: Subgroup analysis for death or severe bilateral hearing loss

	Events/total (%) Dexamethasone	Events/total (%) Placebo	OR (95% CI)	p	Test for heterogeneity between studies χ^2	df	p
**Consciousness level**							
Normal	52/265 (20%)	53/283 (19%)	1·02 (0·65–1·57)	0·95	4·07	5	0·54
Mild impairment	96/317 (30%)	111/322 (34%)	0·82 (0·57–1·17)	0·28	2·75	5	0·74
Moderate impairment	105/225 (47%)	107/201 (53%)	0·79 (0·53–1·20)	0·27	10·35	5	0·07
Severe impairment	87/129 (67%)	92/126 (73%)	0·68 (0·38–1·20)	0·18	4·91	5	0·43
**Subtotal (I^2=0·0%, p=0·715)**			**0·83 (0·67–1·03)**				
**CSF white cell count (cells per µL)**							
0–99	48/71 (68%)	36/57 (63%)	1·29 (0·59–2·82)	0·52	5·17	5	0·40
100–999	123/254 (48%)	128/254 (50%)	0·91 (0·63–1·32)	0·62	2·19	5	0·82
1000–9999	109/426 (26%)	132/453 (29%)	0·82 (0·60–1·12)	0·22	6·93	5	0·23
≥10 000	53/170 (31%)	59/153 (39%)	0·72 (0·44–1·18)	0·19	2·41	5	0·79
**Subtotal (I^2=0·0%, p=0·635)**			**0·85 (0·69–1·05)**				
**CSF glucose**							
<20 mg	246/595 (41%)	267/583 (46%)	0·82 (0·65–1·05)	0·12	7·18	5	0·21
≥20 mg	57/271 (21%)	55/269 (20%)	0·99 (0·64–1·54)	0·97	3·84	5	0·57
**Subtotal (I^2=0·0%, p=0·460)**			**0·86 (0·69–1·06)**				
**CSF protein**							
<250 mg	54/264 (20%)	45/233 (19%)	1·10 (0·69–1·74)	0·69	2·22	5	0·82
≥250 mg	227/543 (42%)	247/563 (44%)	0·89 (0·69–1·15)	0·37	6·99	5	0·22
**Subtotal (I^2=0·0%, p=0·432)**			**0·94 (0·75–1·17)**				
**Pyogenic organism seen on microscopy**							
No	100/333 (32%)	99/293 (34%)	0·88 (0·61–1·27)	0·50	3·90	5	0·56
Yes	232/598 (39%)	253/614 (41%)	0·87 (0·68–1·11)	0·27	13·64	5	0·02
**Subtotal (I^2=0·0%, p=0·959)**			**0·87 (0·71–1·07)**				
**Causative organism**							
Unknown	63/162 (39%)	54/151 (36%)	1·30 (0·77–2·19)	0·32	5·99	5	0·31
S pneumoniae	170/355 (48%)	181/349 (52%)	0·79 (0·58–1·08)	0·14	8·76	5	0·12
N meningitidis	12/115 (10%)	12/110 (11%)	0·93 (0·40–2·16)	0·87	4·12	4	0·39
H influenzae	37/125 (30%)	54/149 (36%)	0·73 (0·43–1·23)	0·23	2·24	3	0·52
S suis	5/68 (7%)	15/65 (23%)	0·12 (0·01–2·43)	0·01	–	–	–
Aerobic gram-negative bacilli	33/46 (72%)	22/31 (71%)	1·53 (0·44–5·29)	0·50	0·28	2	0·87
Other	11/49 (22%)	16/63 (25%)	0·58 (0·22–1·53)	0·23	4·20	4	0·38
Definitely not BM	12/22 (55%)	9/16 (56%)	1·06 (0·28–4·03)	0·93	1·22	1	0·27
**Subtotal (I^2=0·0%, p=0·510)**			**0·86 (0·69–1·07)**				
**BM confirmation**							
Confirmed	268/758 (35%)	300/767 (39%)	0·82 (0·66–1·03)	0·08	8·88	5	0·11
Probable	63/162 (39%)	54/151 (36%)	1·30 (0·77–2·19)	0·32	5·99	5	0·31
**Subtotal (I^2=60·4%, p=0·112)**			**0·88 (0·72–1·08)**				

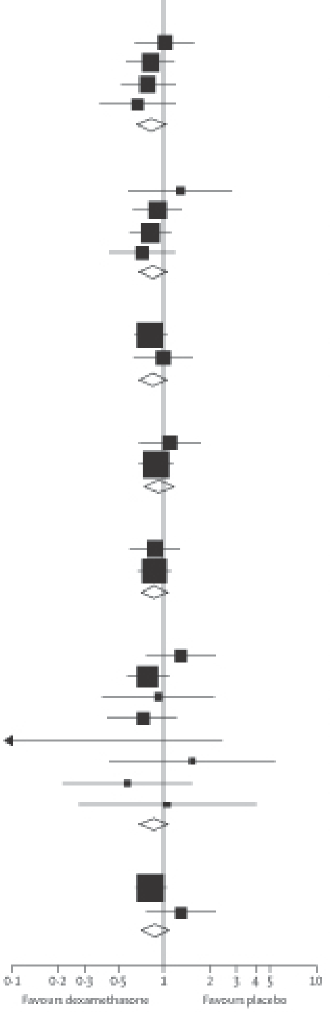

BM=bacterial meningitis. OR=odds ratio.

Webfigure 4a: Subgroup analysis for any hearing loss in survivors

	Events/total (%)		OR (95% CI)	p	Test for heterogeneity between studies		
	Dexamethasone	Placebo			χ^2	df	p
**Age (years)**							
<5	46/182 (24%)	67/207 (32%)	0·65 (0·42–1·02)	0·06	0·11	2	0·95
5–15	20/62 (32%)	17/55 (31%)	1·25 (0·55–2·81)	0·59	1·30	1	0·25
15–55	67/323 (21%)	84/316 (26%)	0·77 (0·53–1·12)	0·17	0·08	2	0·94
>55	28/93 (30%)	27/79 (34%)	0·69 (0·34–1·43)	0·32	0·39	2	0·82
**Subtotal (I²=0·0%, p=0·577)**			**0·75 (0·59–0·97)**				
**Sex**							
Female	72/270 (27%)	81/247 (32%)	0·78 (0·53–1·15)	0·21	3·27	5	0·64
Male	90/402 (22%)	114/412 (28%)	0·77 (0·56–1·07)	0·11	2·38	5	0·79
**Subtotal (I²=0·0%, p=0·961)**			**0·77 (0·60–0·99)**				
**Preadmission symptoms <48 h**							
No	81/303 (27%)	116/328 (35%)	0·70 (0·49–0·99)	0·04	0·76	4	0·94
Yes	79/358 (22%)	77/321 (24%)	0·90 (0·62–1·30)	0·58	0·61	5	0·99
**Subtotal (I²=0·0%, p=0·335)**			**0·79 (0·61–1·02)**				
**Malnutrition**							
Likely normal	92/468 (20%)	124/469 (26%)	0·70 (0·51–0·96)	0·03	0·40	4	0·98
Likely malnutrition	70/204 (34%)	71/191 (36%)	0·89 (0·59–1·36)	0·60	1·67	4	0·81
**Subtotal (I²=0·0%, p=0·369)**			**0·76 (0·59–0·98)**				
**HIV status**							
Likely negative	110/511 (22%)	140/503 (28%)	0·73 (0·54–0·99)	0·04	0·53	5	0·99
Likely positive	41/117 (35%)	43/114 (37%)	0·93 (0·54–1·60)	0·79	0·15	1	0·73
**Subtotal (I²=0·0%, p=0·445)**			**0·77 (0·59–1·01)**				
**Active antibiotic before first dose**							
No	111/473 (23%)	118/455 (26%)	0·91 (0·67–1·23)	0·54	2·13	5	0·83
Yes	50/195 (26%)	71/192 (37%)	0·57 (0·36–0·89)	0·01	1·12	4	0·87
**Subtotal (I²=64·7%, p=0·093)**			**0·79 (0·61–1·01)**				
**Heart rate (beats per min)**							
<120	79/410 (19%)	115/405 (28%)	0·63 (0·45–0·88)	0·01	2·23	5	0·82
≥120	81/249 (33%)	77/247 (31%)	1·02 (0·69–1·51)	0·92	3·41	5	0·64
**Subtotal (I²=70·2%, p=0·067)**			**0·77 (0·60–1·00)**				
**Blood haemoglobin**							
<10 g/dL	54/204 (26%)	74/209 (35%)	0·65 (0·42–0·99)	0·05	2·89	5	0·76
≥10 g/dL	95/433 (22%)	102/408 (25%)	0·86 (0·62–1·20)	0·38	3·38	5	0·64
**Subtotal (I²=2·7%, p=0·311)**			**0·77 (0·60–1·01)**				

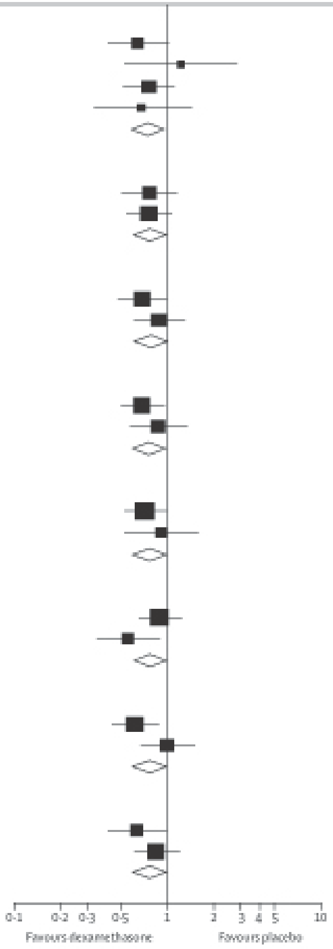

OR=odds ratio.

Webfigure 4b: Subgroup analysis for any hearing loss in survivors

	Events/total (%)		OR (95% CI)	p	Test for heterogeneity between studies		
	Dexamethasone	Placebo			χ^2	df	p
**Consciousness level**							
Normal	40/232 (17%)	51/258 (20%)	0·83 (0·52–1·33)	0·44	2·68	5	0·75
Mild impairment	65/250 (26%)	89/244 (36%)	0·63 (0·43–0·94)	0·02	3·88	5	0·57
Moderate impairment	43/136 (32%)	38/114 (33%)	0·97 (0·54–1·74)	0·93	1·45	5	0·92
Severe impairment	14/53 (27%)	17/42 (40%)	0·53 (0·21–1·33)	0·17	2·85	5	0·72
**Subtotal (I^2=0·0%, p=0·404)**			**0·73 (0·57–0·95)**				
**CSF white cell count (cells per μL)**							
0–99	14/29 (48%)	7/23 (30%)	2·58 (0·75–8·87)	0·12	4·43	3	0·22
100–999	47/153 (31%)	46/150 (31%)	1·03 (0·62–1·70)	0·90	4·26	5	0·51
1000–9999	68/346 (20%)	107/363 (29%)	0·60 (0·42–0·86)	0·004	2·24	5	0·84
≥10 000	29/131 (22%)	29/112 (26%)	0·88 (0·48–1·61)	0·67	2·92	5	0·71
**Subtotal (I^2=0·0%, p=0·911)**			**0·79 (0·61–1·02)**				
**CSF glucose**							
<20 mg	109/405 (27%)	124/378 (33%)	0·77 (0·56–1·05)	0·1	0·80	5	0·97
≥20 mg	42/223 (19%)	58/234 (25%)	0·74 (0·46–1·17)	0·2	4·31	5	0·51
**Subtotal (I^2=0·0%, p=0·890)**			**0·76 (0·59–0·99)**				
**CSF protein**							
<250 mg	44/228 (19%)	56/208 (27%)	0·68 (0·43–1·09)	0·11	6·69	5	0·24
≥250 mg	95/359 (26%)	108/368 (29%)	0·88 (0·63–1·22)	0·44	2·30	5	0·83
**Subtotal (I^2=0·0%, p=0·376)**			**0·81 (0·62–1·06)**				
**Pyogenic organism seen on microscopy**							
No	35/226 (15%)	39/210 (19%)	0·79 (0·47–1·32)	0·36	3·58	5	0·61
Yes	124/426 (29%)	147/429 (34%)	0·81 (0·60–1·10)	0·17	0·51	5	0·99
**Subtotal (I^2=0·0%, p=0·935)**			**0·80 (0·62–1·05)**				
**Causative organism**							
Unknown	15/107 (14%)	19/106 (18%)	0·85 (0·41–1·76)	0·66	5·42	5	0·37
S pneumoniae	68/224 (30%)	66/204 (32%)	0·91 (0·59–1·41)	0·68	2·69	5	0·76
N meningitidis	11/111 (10%)	18/106 (17%)	0·56 (0·25–1·26)	0·15	3·74	4	0·44
H influenzae	20/97 (21%)	33/112 (29%)	0·65 (0·34–1·27)	0·20	3·59	5	0·61
S suis	36/68 (53%)	39/62 (63%)	0·66 (0·33–1·35)	0·25	..	..	..
Aerobic gram-negative bacilli	2/15 (13%)	9/14 (64%)	0·17 (0·02–1·26)	0·05	1·73	2	0·42
Other	8/40 (20%)	10/49 (20%)	0·90 (0·30–2·72)	0·86	3·09	3	0·38
Definitely not BM	2/10 (20%)	1/7 (14%)	0·86 (0·05–15·10)	0·92	..	..	..
**Subtotal (I^2=0·0%, p=0·806)**			**0·75 (0·58–0·98)**				
**BM confirmation**							
Confirmed	145/555 (26%)	175/547 (32%)	0·78 (0·60–1·02)	0·07	0·90	5	0·96
Probable	15/107 (14%)	19/106 (18%)	0·85 (0·41–1·76)	0·66	5·42	5	0·37
**Subtotal (I^2=0·0%, p=0·828)**			**0·79 (0·61–1·01)**				

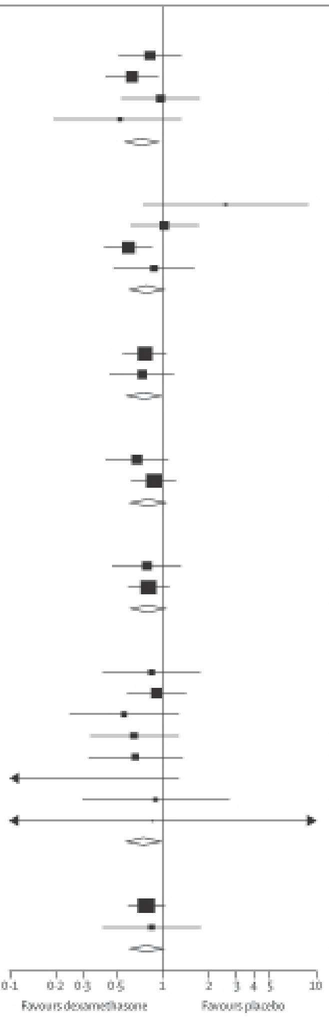

BM=bacterial meningitis. OR=odds ratio.

Webtable 1 – Analysis restricted to sites in which ceftriaxone was given (Vietnam, Malawi adults and Latin America)

	**DXM: events/total**<br>**PLA: events/total**	**Odds Ratio**<br>**(95% CI)**<br>**2P**	**Test for homogeneity between studies**<br>**P**<br>**chi-sq (dof)**
Death	167/557 (30%)<br>165/573 (29%)	1.06 (0.81-1.41)<br>2P=0.66	P=0.58<br>chi2(3)=1.97
Death or severe neurology or bilateral severe deafness	218/543 (40%)<br>243/559 (43%)	0.86 (0.67-1.11)<br>2P=0.24	P=0.72<br>chi2(3)=1.34
Death or any neurology sequelae or any hearing loss	324/542 (60%)<br>343/559 (61%)	0.94 (0.73-1.12)<br>2P=0.60	P=0.71<br>chi2(3)=1.39
Death or severe bilateral hearing loss	187/388 (36%)<br>201/536 (38%)	0.89 (0.68-1.17)<br>2P=0.42	P=0.40<br>chi2(3)=2.94
Any deafness (survivors)	100/357 (28%)<br>125/372 (34%)	0.76 (0.55-1.05)<br>2P=0.09	P=0.97<br>chi2(3)=0.26

Webtable 2 - Logistic regression

	**Effect of dexamethasone : odds ratios(95% confidence interval) multiple logistic regression**					
**ALL stratified by study**	**Death**			**Death or severe hearing loss**		
Adjustment	HIV -	HIV +	ALL	HIV -	HIV+	ALL
Unadjusted	1.01 (0.76-1.36)	0.97 (0.70-1.34)	0.97 (0.78-1.19)	0.84 (0.65-1.09)	0.97 (0.68-1.38)	0.89 (0.73-1.08)
Age**	1.01 (0.76-1.36)	0.97 (0.70-1.34)	0.97 (0.78-1.19)	0.84 (0.65-1.09)	0.98 (0.69-1.40)	0.89 (0.73-1.08)
Consciousness*	0.96 (0.91-1.04)	0.95 (0.7-1.3)	0.92 (0.74-1.14)	0.81 (0.62-1.06)	0.94 (0.65-1.35)	0.85 (0.7-1.04)
Pre-admission symptoms**	1.01 (075-1.37)	0.97 (0.70-1.35)	0.95 (0.77-1.18)	0.85 (0.68-1.11)	0.98 (0.68-1.40)	0.89 (0.72-1.09)
Age+consciousness*	0.97 (070-1.34)	0.95 (0.68-1.34)	0.91 (0.73-1.15)	0.78 (0.59-1.03)	0.94 (0.65-1.4)	0.83 (0.67-1.03)
Age + pre-admission**	1.02 (0.75-1.38)	0.97 (0.70-1.35)	0.96 (0.78-1.19)	0.85 (0.65-1.11)	0.98 (0.69-1.40)	0.89 (073-1.09)
Consciousness*+pre-admission symptoms	0.96 (0.70-1.38)	0.95 (0.67-1.33)	0.92 (0.73-1.15)	0.77 (0.59-1.02)	0.93 (0.65-1.35)	0.83 (0.67-1.02)
Age+consciousness*+pre-admission symptoms**	0.99 (0.71–1.38)	0.95 (0.67 – 1.34)	0.91 (0.72-1.14)	0.80 (0.60-1.07)	0.95 (0.65-1.37)	0.85 (0.68-1.05)

* Conciousness was divided into 4 groups and treated as a continuous variable

**Pre-admission symptoms greater or less than 48 hours

***Age was a continuous variable.

Webtable 3 - Results restricted to patients where HIV status is measured. [No patient in the European or Latin American studies were tested for HIV]

	HIV definite positive			HIV definite negative		
	DXM: events/total<br>PLA: events/total	Odds Ratio<br>(95% CI)<br>2P	Test for homogeneity between studies<br>P<br>chi-sq (dof)	DXM: events/total<br>PLA: events/total	OR (95% CI)<br>2P	Test for homogeneity between studies<br>P<br>chi-sq (dof)
Death	145/269 (54%)<br>153/280 (55%)	0.97 (0.70- 1.36)<br>2P=0.87	P=0.073<br>chi2(2)=5.23	84/394 (21%)<br>69/379 (18%)	1.20 (0.83-1.74)<br>2P=0.32	P=0.15<br>chi2(2)=3.86
Death or severe neurology or bilateral severe deafness	178/260 (68%)<br>183/271 (68%)	1.06 (0.73-1.54)<br>2P=0.74	P=0.47<br>chi2(2)=1.50	150/396 (38%)<br>142/383 (37%)	0.99 (0.73-1.36)<br>2P=1.0	P=0.08<br>chi2(2)=5.14
Death or any neurology sequelae or any hearing loss	199/260 (77%)<br>205/271 (76%)	1.06 (0.71-1.58)<br>2P=0.77	P=0.50<br>chi2(2)=1.45	214/396 (54%)<br>215/383 (56%)	0.9 (0.69-1.20)<br>2P=0.50	P=0.28<br>chi2(2)=2.54
Death or severe bilateral hearing loss	162/255 (64%)<br>169/263 (64%)	0.98 (0.69-1.41)<br>2P=0.93	P=0.26<br>chi2(2)=2.69	111/371 (30%)<br>107/357 (30%)	0.96 (0.69-1.33)<br>2P=0.79	P=0.082<br>chi2(2)=5
Any deafness (survivors)	39/110 (35%)<br>42/111 (38%)	0.92 (0.53-1.60)<br>2P=0.78	P=0.72<br>chi2(1)=0.13	79/287 (28%)<br>98/288 (34%)	0.74 (0.52-1.06)<br>2P=0.01	P=0.85<br>chi2(2)=0.33
